# Molecular Targets in Hepatocarcinogenesis and Implications for Therapy

**DOI:** 10.3390/jcm7080213

**Published:** 2018-08-13

**Authors:** Meng-Yu Wu, Giuo-Teng Yiang, Pei-Wen Cheng, Pei-Yi Chu, Chia-Jung Li

**Affiliations:** 1 Department of Emergency Medicine, Taipei Tzu Chi Hospital, Buddhist Tzu Chi Medical Foundation, New Taipei City 231, Taiwan; skyshangrila@gmail.com (M.-Y.W.); gtyiang@gmail.com (G.-T.Y.); 2 Department of Emergency Medicine, School of Medicine, Tzu Chi University, Hualien 970, Taiwan; 3 Yuh-Ing Junior College of Health Care & Management, Kaohsiung 807, Taiwan; pwcheng@vghks.gov.tw; 4 Department of Medical Education and Research, Kaohsiung Veterans General Hospital, Kaohsiung 813, Taiwan; 5 School of Medicine, College of Medicine, Fu Jen Catholic University, New Taipei City 231, Taiwan; 6 Department of Pathology, Show Chwan Memorial Hospital, Changhua 500, Taiwan; 7 National Institute of Cancer Research, National Health Research Institutes, Miaoli 704, Taiwan; 8 Research Assistant Center, Show Chwan Memorial Hospital, Changhua 500, Taiwan

**Keywords:** liver cancer, hepatocarcinogenesis, inflammasome, NF-κB

## Abstract

Hepatocarcinogenesis comprises of multiple, complex steps that occur after liver injury and usually involve several pathways, including telomere dysfunction, cell cycle, WNT/β-catenin signaling, oxidative stress and mitochondria dysfunction, autophagy, apoptosis, and AKT/mTOR signaling. Following liver injury, gene mutations, accumulation of oxidative stress, and local inflammation lead to cell proliferation, differentiation, apoptosis, and necrosis. The persistence of this vicious cycle in turn leads to further gene mutation and dysregulation of pro- and anti-inflammatory cytokines, such as interleukin (IL)-1β, IL-6, IL-10, IL-12, IL-13, IL-18, and transforming growth factor (TGF)-β, resulting in immune escape by means of the NF-κB and inflammasome signaling pathways. In this review, we summarize studies focusing on the roles of hepatocarcinogenesis and the immune system in liver cancer. In addition, we furnish an overview of recent basic and clinical studies to provide a strong foundation to develop novel anti-carcinogenesis targets for further treatment interventions.

## 1. Introduction

Liver cancer constitutes a global health problem, with liver neoplasms representing the second-most frequent cause of cancer-related death in 2012. Moreover, the incidence rate has been recently increasing, with estimates as high as 1.9 to 4.3 cases/100,000 individuals in males and 0.8 to 13.9 cases/100,000 in females [1]. These significant incidence and mortality rates have led to families and society bearing extensive burden for associated medical care and health insurance, often leading to financial crises. Notably, it is estimated that 70% to 90% of primary liver tumors comprise of hepatocellular carcinoma (HCC), the most common liver cancer. Several studies have reported risk factors of HCC, which include chronic liver cirrhosis, hepatitis B virus (HBV) infection, hepatitis C virus (HCV) infection, alcohol abuse, metabolic syndrome, and nonalcoholic fatty liver disease [2,3,4]. Many cofactors such as environmental toxins have also been investigated; they include smoking, intake of aflatoxin (odds ratio: 3.04; 95% confidence interval: 1.11–8.30), drinking pond-ditch water, and chewing betel nut (odds ratio: 3.49; 95% confidence interval: 1.74–6.96) [5,6,7,8,9]. With aflatoxin exposure, mutation of the p53 tumor suppressor gene has been reported in patients with HCC; similar results were also noted in cases of HBV exposure, which may involve the same pathway in hepatocarcinogenesis [10,11,12]. However, despite numerous years of investigations from bench to bedside, the detailed mechanism of hepatocarcinogenesis is still unclear. We consider that a clear pathogenesis might provide a strong foundation for early diagnosis and therapeutic intervention. Toward this end, this article provides a detailed overview of basic and clinical studies of molecular regulation and cell biology in hepatocarcinogenesis and summarizes the current understanding of mechanisms to furnish new therapeutic targets and strategies for liver cancer.

## 2. Pathogenesis of Hepatocellular Carcinoma

Hepatocarcinogenesis constitutes multiple, complex steps that usually involve several pathways, including telomere dysfunction, cell cycle, WNT/β-catenin signaling, oxidative stress and mitochondria dysfunction, autophagy, apoptosis, and AKT/mTOR signaling. After exposure to HBV infection, HCV infection, alcohol abuse, metabolic syndrome, environmental toxins, or nonalcoholic fatty liver disease, chronic liver injury can lead to gene mutation, accumulation of oxidative stress, and local inflammation. During the natural course of HCC development, the cirrhotic liver may persist for over 10 years and develop into low-grade dysplastic nodules (LGDNs), which comprise cirrhotic nodules with low-grade dysplasia. After five to seven years’ follow-up, the low-grade dysplastic nodules may shift to high-grade dysplastic nodules (HGDNs), which can develop into early-stage (stages 0 and A) and advanced HCC (stages B and C). The duration and prognosis of malignant transformation vary according to cell type and underlying liver disease [13]. In early hepatocarcinogenesis, somatic genomic alterations play a critical role in development, progression, distance of metastasis, and even resistance. They also promote HCC proliferation, survival, invasion, and inflammation [14,15,16]. Current research has been devoted to understanding the detailed pathways within the enormous complexity of hepatocarcinogenesis. In particular, several pathways of oncogenesis have been reported, such as telomere maintenance and WNT/β-catenin signaling, receptor tyrosine kinase, angiogenesis, transforming growth factor (TGF)-β, Janus kinase (JAK)/STAT, and ubiquitin proteasome pathways. In the following sections, we discuss the pathways of oncogenesis that play an important role in HCC.

## 3. Telomere Maintenance

**Figure 1:**
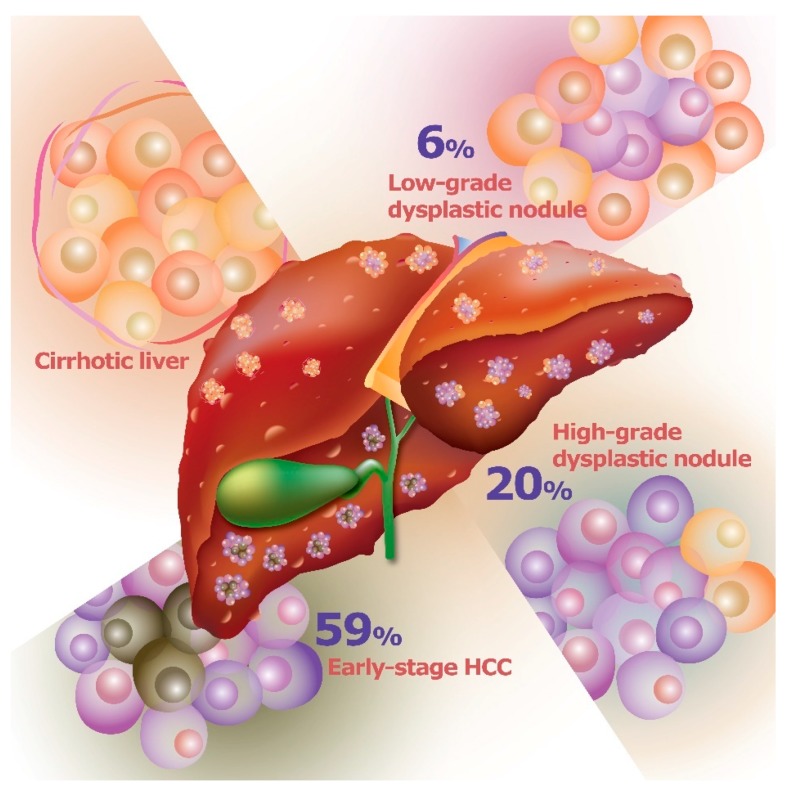
Telomerase reverse transcriptase (TERT) mutation prevalence during HCC progression.

Telomeres, regions of repetitive nucleotide sequences at the ends of chromosomes, protect chromosomes against end-to-end fusion via telomerase, which is an enzyme comprised of telomerase reverse transcriptase (TERT) and the telomerase RNA component (TERC). Telomerase regulates the length of telomeres to control chromosomal stability, leading to cell proliferation, survival, or death. In mature cells, telomerase is usually suppressed to inhibit cell proliferation because as DNA polymerase cannot replicate terminal nucleotide sequences, the telomeres generally become shorter after cell divisions. Conversely, after exposure to several risk factors, the resulting chronic liver injury increases cell turnover rate and leads to telomere shortening, which also induces hepatocyte apoptosis [17,18,19]. Cell death in turn causes the liver to lose its normal architecture and activates liver fibrosis and cirrhosis. In addition, the telomere-shortening effect may cause chromosomal instability and trigger oncogenesis. Notably, mutations in the *TERT* gene represent the most common mutation in HCC, leading to increased *TERT* transcript expression Accordingly, many studies have investigated telomerase complex dysfunction, which promotes the development of hepatocarcinogenesis [20,21]. In an animal study, the deficiency in telomerase RNA component could promote the development of cirrhosis and HCC [22]. Moreover, in a human study, re-expression of TERT was noted in LGDNs and HGDNs, which suggests its involvement in hepatocarcinogenesis. In particular, these activating mutations are identified as about 6% of LGDNs, 19% of HGDNs, 61% of early HCC, and 42% of progressed HCC [15,23,24,25] (Figure 1).

## 4. HBV and HCV Infections

HBV and HCV comprise of two main hepatitis viruses associated with hepatocarcinogenesis. HBV, a partially double-stranded DNA virus, encodes a number of viral proteins, including a reverse transcriptase/DNA polymerase, capsid protein, L, M, S envelope proteins, and protein x [26]. The transformation process of HBV may induce DNA microdeletions to trigger hepatocarcinogenesis [27,28]. Numerous studies have revealed the association between DNA microdeletions and oncogenes, such as TERT, platelet-derived-growth-factor-β (PDGF-β) or receptor-β (PDGFR-β), and mitogen activated protein kinase 1 (MAPK 1). The HBx protein also activates the expression of growth-control genes during transcription, such as those encoding SRC tyrosine kinases, Ras, Raf, MAPK, ERK, and JNK, and inhibits the tumor suppressor gene, *p53*, to promote cell proliferation [29,30,31,32]. In animal studies, HBx exhibited significant potentiality for the development of HCC [33,34]. In addition, the immune responses to HBV have been shown to constitute another key factor in hepatocarcinogenesis. To combat viral infection, the resulting severe systemic and local inflammation leads to accelerated hepatocyte apoptosis, necrosis, and regeneration, causing carcinogenesis [26,35,36,37]. In chronic HBV infection, the course from hepatocyte apoptosis and necrosis, through local inflammation, to liver regeneration, becomes a vicious circle that can activate hepatocarcinogenesis. 

HCV, a non-cytopathic virus with a positive-stranded RNA genome encoding several non-structural proteins including NS2, NS3, NS4A, NS5A, and NS5B, is unable to integrate into the host genome [38,39,40]. Thus, HCV induces hepatocarcinogenesis by several indirect mechanisms. HCV usually induces a chronic infection, leading to a vicious cycle of hepatocarcinogenesis (i.e., 10% in HBV infection versus 60% in HCV infection). The severe local inflammation and high rates of replication trigger the accumulation of DNA mutations and telomere shortening, which might lead to immune evasion. Numerous data have confirmed that HCV exhibits substantial propensity for liver cirrhosis, which constitutes a significant risk factor for hepatocarcinogenesis. In addition, the promotion of hepatocarcinogenesis via the immune responses of HCV infection has been revealed through a transgenic skin-tumor model, which indicates that deficiencies of pro-inflammatory T cells may decrease the incident rate of tumor development. However, the detailed pathophysiology of immune-mediated hepatocarcinogenesis remains unclear. In current models, it is considered that the HCV RNA and core proteins, especially the NS5A non-structural protein, could impair the activity of dendritic cells and decrease the functions of T-cells.

## 5. Signaling Pathways in the Tumor Microenvironment

**Figure 2:**
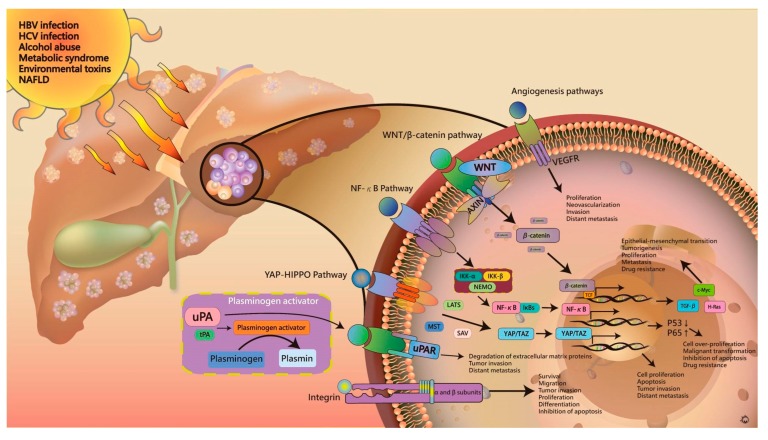
Detailed mechanisms in hepatocarcinogenesis. The exposure to hepatotoxic agents triggers gene mutation and local inflammation via Wnt/β-catenin, NF-κB, signaling, the YAP-HIPPO pathway, and angiogenesis pathways. PA and integrin are also involved in the carcinogenesis process.

After exposure to hepatotoxic agents, the resulting gene mutations and local inflammation induce a number of cellular signaling pathways to cause dysregulation of hepatocellular proliferation and cellular dysfunction. Numerous signaling pathways of hepatogenesis have been reported, which include Wnt/β-catenin, NF-κB, YAP-HIPPO, and angiogenesis pathways. Plasminogen activator and integrin are also involved in the development of liver cancer. In the following sections, we describe the current concept of common pathways and mechanisms of hepatogenesis. The details of these pathways can improve our understanding of HCC pathogenesis and provide novel drug targets for clinical research and application (Figure 2).

### 5.1. WNT/β-Catenin Signaling Pathway

The WNT/β-catenin signaling pathway regulates embryogenesis, cell differentiation, proliferation, and tumorigenesis, and constitutes one of the common pathways in HCC. This signaling pathway is also involved in many processes, such as gene expression regulation, cell migration and adhesion, and cell polarization. In 3% to 16% of HCC, mutations were noted in inhibitor genes of the WNT pathway, such as *AXIN1* and *AXIN* 2, in addition to inactivation of the tumor suppressor gene adenomatous polyposis coli (*APC*), leading to activation of the WNT pathway. Following the activation of WNT signaling, β-catenin is accumulated and translocated to activate downstream target genes via T-cell factor (TCF)/lymphoid enhancer factor (LEF).

In recent years, the epithelial-mesenchymal transition (EMT) has been considered as the core mechanism involved in tumorigenesis, proliferation, metastasis, and drug resistance. The WNT/β-catenin signaling pathway is considered to be the core signal that regulates EMT. Studies have found that 20% to 90% of liver cancers exhibit abnormal activation of the WNT/β-catenin signaling pathway induced by multiple mechanisms [41]. For example, in a transgenic mouse model, it was found that β-catenin accumulated in the nucleus accompanied with high expression of c-Myc and TGF-β. Moreover, when β-catenin and H-Ras proteins are mutated simultaneously, the incidence of liver cancer in mice reaches 100% [42]. Thus, abnormal activation of the WNT/β-catenin signaling pathway constitutes one of the direct causes of the onset of liver cancer. This pathway also plays an important role in many different tumors, including gastric cancer, colorectal cancer, lung cancer, and other high-grade tumors, and is therefore a hot topic in tumor pathology and targeted drug research [43].

### 5.2. NF-κB Pathway

Recent studies have indicated that the positive expression rate of NF-κB detected in human HCC can reach >70%, with a statistically significant difference between positive expression rate in HCC and normal liver tissue. This result demonstrates that NF-κB plays an important role in the progression of liver cancer [44]. Moreover, once the cells are exposed to a stressful environment, NF-κB translocates into the nucleus and binds to the NF-κB binding site on the Cyclin D1 promoter, causing cell cycle progression from G1 to S-phase resulting in cell over-proliferation. Malignant transformation may also occur under other concomitant factors such as the occurrence of oncogenic P53, which under normal conditions has a surveillance and regulatory role with regard to DNA damage during the cell cycle in G0/G1 phase cells [45]. In addition, NF-κB can inhibit the activity of P53, which may protect malignant cells against apoptosis and promote the formation of tumors. Similarly, the expression of NF-κB p65 is also significantly increased in patients with HCC. The astrocyte elevated gene (AEG) has also been recently found to exhibit a high correlation with the occurrence of liver cancer, with the expression of AEG and NF-κB p65 in liver cancer tissue being significantly higher than that of adjacent as well as normal liver tissue. AEG may pass through with NF-κB p65. Interaction of these proteins induces the activation of the NF-κB pathway, leading to the occurrence and progression of liver cancer and further affecting the prognosis of patients [46].

The rapid proliferation of tumor cells is an extremely energy-consuming process. Even when oxygen supply is sufficient, glycolysis still represents the main mode of metabolism to continuously provide oxygen and energy for cell growth and proliferation. The NF-κB family member RelA plays an important role in the regulation of mitochondrial function. In addition, a unique DNA-binding domain in NF-κB p65 underlies its function of inhibiting P53, which can modulate the energy supply and metabolic balance of tumor cells by regulating glycolysis and aerobic respiration [47]. In particular, hepatocarcinoma cells proliferate rapidly but are unable to generate sufficient blood supply, leading to hypoxia in the tumor tissue. The resulting aerobic glycolysis of hepatic tumor cells can produce large amounts of lactic acid, causing the pH of the tumor tissue to decrease [48,49]. In turn, the low pH microenvironment can also affect a variety of physiological activities of cells to further enhance the ability of metastasis and invasion of tumor cells [50]. Notably, previous studies have shown that the activation of NF-κB can protect tumor cell activity while enhancing its proliferation and resistance to the influence of drugs and radioactive substances to promote the development and spread of liver cancer [51]. Tumor hypoxia is an effective oxygen reduction state in most solid tumors and can be used as a prognostic factor for poor prognosis. It is also known to play a role in the microenvironment of tumor inflammation by modulating inflammatory mediator signals in cancer and adjacent cells. Hypoxia and inflammation share an interdependent relationship. Contributors to tissue hypoxia during inflammation include an increase in the metabolic demands of cells and a reduction in metabolic substrates [52]. Therefore, the hypoxia factor HIF-1α interacts with the inflammatory factor NF-κB, which confirms the role of hypoxia in innate immunity and inflammation [53]. Tumor hypoxia was identified as an enhancer of inflammation-mediated metastasis. Inflammation caused by hypoxia may play a role in improving the nurturing environment for cancer development [54].

### 5.3. Yes-Associated Protein (YAP)-Hippo Pathway

YAP has a relative molecular weight of about 65 kDa, is rich in prolines, and acts as a multifunctional connexin and transcription coactivator in cells. YAP constitutes a core effector in the Hippo pathway and plays a role in intracellular signaling and transcriptional regulation in cells. The Hippo-YAP signaling pathway regulates the size of liver organs and the proliferation, apoptosis, invasion, and metastasis of hepatoma cells. YAP is expressed to a certain degree in the cytoplasm and nucleus of human epithelial, muscle, and other cells, albeit in the majority of malignant tumor cells, such as liver cancer, cholangiocarcinoma, colorectal cancer, breast cancer, lung cancer, and glioma. YAP is highly expressed, indicating that YAP has a high correlation with the occurrence and development of tumors [55,56]. Numerous studies have found that the transcriptional co-activators TAZ and YAP comprise downstream effectors of the Hippo pathway. The expression levels of both TAZ and YAP in liver cancer are elevated, with the expression of TAZ being relatively high [57]. In patients with high YAP expression, the tumor size is larger with cirrhosis, vascular invasion, and intrahepatic metastasis being more likely to regulate the proliferation, invasion, and apoptosis of hepatocarcinoma cells [58]. In addition, the Hippo-YAP signaling pathway is blocked, and up-regulation of YAP can promote the progression of liver cancer. The high expression of YAP in the liver can also result in hepatomegaly, eventually leading to the formation of liver cancer in mice [59]. YAP overexpression in the mouse liver results in a 2-fold increase in the mass of the liver after 1 week, with the weight of the liver reaching 20% of the weight of the mouse. After 5 months, almost all of the livers progressed to liver cancer. However, when YAP expression returned to normal levels, liver tumors were significantly reduced and the liver parenchyma gradually returned to normal [60]. The above study shows that *YAP* as a human candidate oncogene is closely related to the occurrence of liver cancer, and the Hippo-YAP pathway may constitute an important signaling pathway regulating liver tumor formation.

### 5.4. Angiogenesis Pathways

Angiogenic factors can promote the formation of new blood vessels. The formation of tumor neovascularization creates conditions for the invasion and metastasis of tumors and also provides nutrients for the growth of tumor cells. Once tumor neovascularization is initiated, it tends to proliferate indefinitely and be resistant to modulation by the organism. Liver cancer is mainly a multi-vessel tumor in which the shape of the blood vessels is very irregular. Because the tumor-induced neovascularization is only composed of a layer of endothelial cells, the smooth muscle layer of the vessel wall is fissure-like and the basement membrane is thin and easily broken, allowing the tumor cells to easily enter these blood vessels. In addition, some blood vessels have no obvious lumen, with only single or several endothelial cell masses, thus showing significant heterogeneity. This not only creates very favorable conditions for tumor invasion and metastasis, but also comprises the basis for the sustainable growth of tumor cells [61]. 

Vascular endothelial growth factor (VEGF) is the most important factor for promoting tumor angiogenesis. VEGF expression in liver cancer tissues is significantly higher than that in adjacent tissues as well as that in other liver diseases and normal liver [62]. As the growth of tumors is reliant on the production of blood vessels, high VEGF expression in HCC is closely related to its distant metastasis, formation of tumor thrombus, and postoperative recurrence [61]. Serum VEGF can also be used to deduce patient immune status and indicate tumor metastasis [63,64]. For example, the serum level of VEGF in patients with primary liver cancer is higher than that in patients with liver cirrhosis (*p* < 0.05). High serum expression of VEGF is also closely related to the metastasis and prognosis of HCC, and can be used as an independent predictor of lymphatic metastasis. Moreover, a positive correlation exists between serum VEGF and alpha-fetoprotein (AFP) (*R*^2^ = 0.488, *p* < 0.05). Serum VEGF and AFP predicted primary hepatic carcinomas could be used to predict occurrence of liver cancer [65]. 

### 5.5. Plasminogen Activator (PA)

PA is a serine protease that catalyzes the conversion of inactive plasminogen to plasmin. PA usually has two forms: Urokinase type plasminogen activator (uPA) and tissue type plasminogen activator (tPA). In addition, the vast majority of uPA is involved in the degradation of extracellular matrix proteins and tumor invasion and metastasis. The complex interaction between uPA, uPA inhibitor (PAI), and uPA receptor (uPAR) can participate in the complete degradation of the extracellular matrix (ECM) [66,67]. In a study of the in vitro invasive ability of tumor cells, the invasiveness of malignant tumor cell lines was positively correlated with the expression level of the uPA and uPAR system in tumor cells. Specifically, Pro-uPA secreted by tumor cells binds to uPAR to activate uPA, which then activates plasminogen bound to the cell surface, converting it into plasmin and degrading tumor cell connections, including laminin, fibrin, fibronectin, and other substrates [68]. The interaction between uPAR and uPA continuously increases the proteolytic activity of the cell surface, facilitating tumor cell infiltration and metastasis. However, a competitive inhibition of uPAR occurs between PAI-1 and uPA, with the formation of the PAI/uPAR complex able to reduce the activity of uPA [69,70]. Accordingly, uPA, PAI-1, and uPAR were confirmed to exert mutual regulatory roles in the invasion and metastasis of hepatoma cells [71]. Recently, researchers have used in situ hybridization and immunohistochemistry to discover that uPA and uPAR are highly expressed in liver cancer tissues, demonstrating that the expression of uPA and uPAR is negatively correlated with the prognosis and survival of patients with liver cancer [72,73].

### 5.6. Integrin

The integrin family comprises of cell surface glycoprotein receptors whose ligands are components of the ECM. Each receptor is a heterodimeric protein consisting of α and β subunits connected by non-covalent bonds. At present, 14 α and 8 β subunits have been identified. However, as the genes of the α and β subunits can be denatured and spliced during transcription, the free combination of α and β subunits can form more than 100 types of heterodimer integrins, forming a huge family. To date, integrins have been confirmed in at least 20 species [74,75]. The combination of ligand/receptor diversity can cause specific changes in the adhesion of tumor cells, affect the migration of tumor cells, and ultimately cause distant metastasis of the tumor [74,76]. The application of molecular cloning, immunohistochemistry, and monoclonal antibody technology has demonstrated that integrins play two major roles during carcinogenesis, development, invasion, and metastasis. Integrins mediate the transfer of information from the ECM into the cell, with this information usually further influencing cell growth and differentiation. Conversely, the transmission of abnormal information can promote the uncontrolled growth and dedifferentiation of tumor cells. Moreover, integrin mediates the adhesion of the ECM and tumor cells. They can regulate the biological function of the matrix by changing its composition, promoting the tumor cells to break away from the primary tumor and penetrate the ECM and the blood vessel walls in the circulatory system. Therein, integrin interacts with white blood cells and platelets, and enables tumor cells to evade host immune surveillance, promoting the formation of metastatic foci of tumor cells in target organs [77]. 

Owing to the complexity and diversity of the structure and function of integrins, their function in liver cancer varies. In addition to the possible loss of integrin subtypes involved in the maintenance of tissue structure and stability, tumor cells acquire a large number of other integrin subtypes that may be involved in tumor cell migration, invasion, and metastasis. Studies have shown that integrin subunits α1, α2, α3, α6, and β1 are distributed on the edge of liver cancer cells. As the connection between liver cancer cells and fibronectin mainly depends on integrin α5β1, this subtype, as a negative regulator of signal transduction, is associated with the malignant phenotype of HCC, with its expression on the poorly differentiated and highly metastatic liver cancer cell membrane being significantly reduced [78,79].

## 6. Farnesoid X Receptor in Hepatocarcinogenesis

The farnesoid X receptor (FXR) is a member of the nuclear receptor family, and plays an important role in the regulation of bile acid metabolism by suppressing the synthesis and import of bile acids in hepatocytes and stimulating their biliary excretion. FXR also regulates glucose and lipid metabolism. FXR activation protects hepatocytes against bile acid-induced cytotoxicity, inhibits hepatic lipogenesis, and increases insulin sensitivity [80]. In recent years, the role of FXR in various liver diseases has been clarified. Therefore, the role of FXR in liver cancer has received much attention and has gradually become a new research hotspot. Huang et al. performed partial liver resection of mice and found that FXR can promote liver regeneration [81]. Hepatic weight and function are restored by regeneration after liver injury, and repair activity will be stopped accurately after normal liver repair is completed. Long-term chronic liver injury can cause repeated necrosis and regeneration of hepatocytes, which are out of control during hepatocyte regeneration and ultimately leads to carcinogenesis [82]. Deuschle et al. found that FXR expression was abnormally reduced in the liver of patients with HCC [83]. In 2007, FXR knockout (FXR−/−) mice spontaneously developed liver tumors; however, no other tumors were noted after 15 months. In contrast, no liver tumors were observed in wild-type mice of the same age [84]. The use of RNA interference technology in vitro to silence FXR gene can promote the growth, metastasis and invasion of Huh7 liver cancer cells [85]. In addition to hepatocellular carcinoma, significant hepatocyte apoptosis and necrosis were observed in the liver of FXR−/− mice, and the expression of cell cycle-associated proteins CyclinD1 and CyclinE1 was increased, indicating that FXR plays an important role in hepatocyte fate [80,86]. Niu et al. found that HBx can interact with FXR and act as a co-activator of FXR. FXR ablation significantly sensitized mice to HBx-induced liver cancer in vivo [87]. In addition, FXR agonist treatment enhances TGF-β induced EMT morphological changes. In contrast, FXR antagonists inhibit the EMT effects induced by TGF-β [80]. These results suggest that FXR deficiency is likely to be one of the important causes of liver cancer induction. The mechanism may be that FXR loss leads to impaired normal liver regeneration, resulting in repeated necrosis and compensatory proliferation of hepatocytes. However, once the self-repair function of hepatocytes is abnormal, it will promote the incidence of HCC.

## 7. Inflammation in Hepatocarcinogenesis

After exposure to acute damage or microbe- or damage-associated microbe patterns (MAMPs or DAMPs) from blood circulation, the liver continuously repairs itself by activating the differentiation and proliferation of hepatocytes. The persistence of cell dysfunction and death triggers the immune response, with chronic liver inflammation causing liver fibrosis and the development of HCC [88]. The concept of necroinflammation has been proposed to explain the mechanism of immune response in HCC. Necroinflammation regulates cell death by necrosis through immunogenic cell death, leading to the release of DAMPs, which activates pro-inflammatory pathways [89,90,91]. The autoamplification loop of necroinflammation finally results in tissue damage and multiple organ dysfunction via increased cellular stress, epigenetic modifications, mitochondrial dysfunction, DNA damage, and genetic instability, which promote hepatocarcinogenesis. The innate and adaptive immune systems play an important role in the detection and elimination of atypical cells. However, in HCC, the immune system is also dysregulated and releases anti-inflammatory cytokines, such as interleukin (IL)-10, IL-13, and TGF-β, to inhibit anti-tumor immune responses.

### 7.1. Inflammasome

Chronic inflammation in tumor microenvironments plays an important role during carcinogenesis in various stages of HCC. Currently, inflammasomes represent a new concept of the innate immune pathway, which lead to liver damage, steatosis, inflammation, and fibrosis by activating pro-inflammatory cytokines IL-1α, IL-1β, and TNFα and inducing pyroptosis [92]. The inflammasome consists of large multiple protein complexes that detect intracellular danger signals via NOD-like receptors (NLR). NLRs belong to the pattern recognition receptor (PRR) family, which consists of several proteins that include NACHT, C-terminal leucin-rich-repeat (LRR), N-terminal CARD, pyrin (PYD), baculoviral inhibitory repeat (BIR), and acidic transactivation domains [93,94,95,96,97,98]. Three subfamilies of proteins are defined according to the NACHT domain: NODs, NLRPs or NALPs, and IPAF [99,100,101,102]. The different inflammasomes are briefly categorized as NLRP1, NLRP3, NLRC4, and AIM2. NLRP3 has been prominently noted in hepatocytes. The inflammasome regulates the immune system by increasing the release and maturation of inflammatory cytokines, such as IL-1β and IL-18, after recognizing pathogen-associated molecular patterns (PAMPs) from viruses, bacteria, tissue injury, and oxidative stress. Inflammasome activation leads to auto-activation into active caspase-1, promoting the activation of IL-1β and IL-18 [103,104,105,106,107]. In turn, these regulate local inflammation including the recruitment of neutrophils and activation of NK cells to produce IFN-γ, and also decrease Th2 cytokines by inhibiting IL-33, a chromatin-associated cytokine [104]. Recently, the release of IL-1β has been suggested to induce pyroptosis [108]. In in vitro studies, the release of IL-1β from Kupffer cells has a critical role in inflammasome mediation of the inflammatory response. High levels of inflammasomes are correlated with liver fibrosis [109,110], with the procarcinogenic functions of inflammasomes inhibiting the anti-metastatic activity of NK and T cells, thereby promoting hepatocarcinogenesis and neoangiogenesis.

### 7.2. NF-κB and STAT3 Signaling in Hepatocarcinogenesis

Hepatic injury resulting from exposure to HBV or HCV infection or toxins may activate resident liver immune cells and recruit circulating immune cells to the liver from the blood, such as Kupffer cells, dendritic cells, T cells, and natural killer T cells, leading to local and systemic immune response. Several studies have confirmed the correlation between inflammation and HCC. In in vitro studies, the NF-κB and STAT3 signaling pathways play an important role in linking the various regulatory pathways of HCC [111]. NF-κB activation promotes cell proliferation, antiapoptosis, angiogenesis, tumor invasion, and distant metastasis in carcinogenesis [112]. The inhibition of NF-κB activity significantly reduces the expression of carcinogenetic growth factors. In the tumor necrosis factor (TNF)-NF-κB axis, inhibition of NF-κB signaling by anti-TNF-α agents or activation of the repressor IkB to control inflammation also inhibits the progression of HCC [113]. Conversely, increasing production of cytokines, such as IL-1β, interferon-γ (IFN-γ), and IL-6, by triggering expression of lymphotoxin heterodimers from T cells and dendritic cells promotes HCC. NF-κB activation triggers the release of chemokines including CXCL10, CXCL1, and CCL7, which attracts monocytes, T cells, B cells, neutrophils, and natural killer (NK) cells. The progression of this inflammatory process favors hepatitis and HCC. In addition, several anti-apoptotic factors including cIAPs, c-FLIP, and BclX are regulated by NF-κB to promote cell proliferation. Moreover, NF-κB activation also increases the rate of drug resistance. Thus, the inhibition of NF-κB activity constitutes an important target of tumor therapy. 

**Figure 3:**
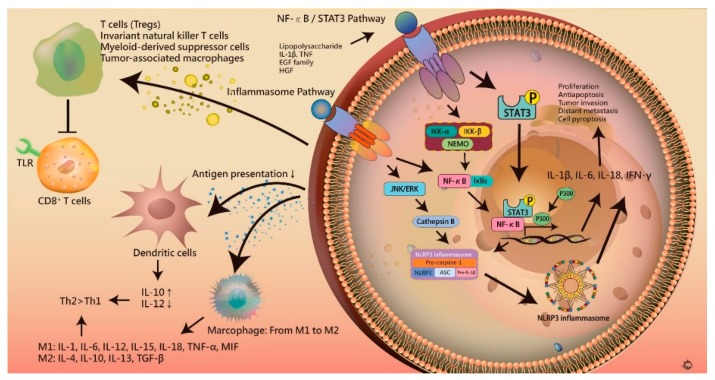
The role of immunity in hepatocarcinogenesis. After liver damage, cell dysfunction and death triggers local and systemic immune responses, leading to liver cirrhosis and hepatocarcinogenesis. Chronic liver inflammation causes the dysregulation of pro- and anti-inflammatory cytokines, such as IL-1β, IL-6, IL-10, IL-12, IL-13, IL-18, and TGF-β, which inhibit anti-tumor immune responses. The activation of NF-κB and STAT3 signaling is also involved. NF-κB also correlates with the formation of inflammasome, leading to the release of IL-1β. The dysregulated cytokines inhibit antigen presentation cells, leading to over-expression of Th2 cytokines, and decrease the activity of CD8+ T cells via T regulatory cells, invariant natural killer T cells, myeloid-derived suppressor cells, and tumor-associated macrophages, causing immune escape.

STAT3 signaling represents another important link connecting inflammation and HCC progression. This is activated by numerous cytokines and growth factors, including IL-6, TNF, the EGF family, and hepatocyte growth factor (HGF) [114,115]. In HBV and HCV infection, the accumulation of oxidative stress from infection and inflammation induces the expression of JNK to activate STAT3. Specifically, the release of cytokines from abnormal autocrine or paracrine signaling stimulates cell surface receptors and regulates STAT3 signaling through the phosphorylation of a critical tyrosine residue (Tyr705), which is mediated by JAKs. Notably, the activation of STAT3 has strong negative feedback from SHP phosphatases and suppressor of cytokine signaling 3 (SOCS3), which is inhibited in tumor cells. In an in vitro study, overexpression of proinflammatory cytokines, such as IL-6, IL-10, IL-11, and TGF-α, increased JAK/STAT3 activity, regulating the liver micro-environment to inhibit apoptosis and promote oncogenic conditions for hepatocarcinogenesis. STAT3 activation has also been reported to be associated with the progression of tumor aggressiveness (Figure 3).

### 7.3. Immune Escape

The liver has multiple functions to maintain normal physiological conditions, including the metabolism of toxins and waste substances, degeneration of blood pathogens, and filtration of pathogens from the gastrointestinal tract via the portal and hepatic systems. Therefore, hepatocytes have developed a unique tolerogenic immune microenvironment to prevent the persistence of local inflammation and autoimmune damage. The innate and adaptive immune responses play an important role in the tolerogenic immune mechanism [116]. In hepatocarcinogenesis, this tolerogenic immune microenvironment allows tumor cells to evade the immune system by several mechanisms. The activity of antigen presentation by antigen presenting cells is decreased owing to low expression of HLA class-I molecules [117]. The accumulation of regulatory T cells (Tregs), a subset of CD4+ T cells, presents anti-tumor immunity to suppress effector CD8 T cells, leading to an ineffectiveness of the immune system [118,119]. Similar results have been reported with regard to invariant natural killer T cells (iNKTs), myeloid-derived suppressor cells (MDSCs), and tumor-associated macrophages (TAMs), along with diminished CD4+ T helper cells [120,121,122,123,124,125]. In macrophages, the phenotypic switch from M1 to M2 phenotype is involved in infectious as well as inflammatory diseases, such as atherosclerosis and carcinogenesis [126,127]. M1 macrophages induce local inflammation by presenting antigens to T cells as antigen presenting cells and releasing pro-inflammatory cytokines including IL-1, IL-6, IL-12, IL-15, and TNF-α. M2 macrophages exert anti-inflammatory functions by releasing Th2 cytokines, such as IL-4, IL-10, and IL-13 [126]. The phenotypic switch increases the activity of Th2 cytokines and promotes hepatocarcinogenesis. A recent study revealed that the deletion of p53 in hepatic stellate cells causes the acceleration of HCC development via M2 polarization, which leads to immune escape. The release of anti-inflammatory cytokines including IL-1, IL-4, IL-5, IL-8, IL-10, TNF, and IFN-γ also allows evasion of the immune system owing to the resultant suppression of immune activity, with the high accumulated level of these cytokines at the liver promoting HCC distant metastasis [128]. Consistent with this, the high level of IL-10 has been correlated with poor clinical outcome in patients with HCC [129,130]. Moreover, inflammatory cells and cytokines demonstrably support cancer stem cells via the IL-6/STAT3 pathway. In vitro, the release of IL-6 may induce expansion of CD133-positive cancer progenitor cells [131]. The IL-6/STAT3 paracrine signaling pathway from TAMs also triggers the proliferation of cancer progenitor cells to facilitate hepatocarcinogenesis [116].

## 8. The Microbiota and HCC

In recent years, the study of intestinal flora imbalance has led to more and more research on related liver diseases. The regulation of probiotic bacteria in intestinal flora may be a new way to treat or prevent the development of liver cancer.

### 8.1. Targeting Probiotics in HCC

Probiotics regulate intestinal flora, permeability, immunity and inflammatory responses, and the intestine and liver are closely linked by hepatic portal vein circulation. Therefore, probiotics play an important role in the treatment of liver diseases [132]. The changes in the number and type of intestinal flora can regulate intestinal flora balance, intestinal inflammation and mucosal barrier function. It can also significantly improve the liver’s hardening and prevent the occurrence of liver cancer. Interestingly, probiotics inhibit endotoxin translocation, while endotoxin is caused by PAMPs and activates associated DAMPs [133]. In addition, probiotics are a safe and inexpensive treatment. Probiotics have special properties, such as stimulation of the immune system, which improves intestinal function by adhering and colonizing intestinal epithelial cells. Moreover, when these strains stimulate the immune system, they must be able to survive and produce considerable health outcomes.

### 8.2. Targeting Gut Microbiota in HCC

Previous studies have pointed out that patients with primary liver cancer are prone to overgrowth of intestinal bacteria, and intestinal flora imbalance will lead to intestinal endotoxemia. Endotoxin, a ligand for TLR4, activates chronic liver function damage, hepatitis, and liver fibrosis through activation of the TLR4 signaling pathway, ultimately promoting the development and progression of HCC [134]. In addition, blocking the TLR4 signaling pathway significantly reduces the development of HCC, which elucidates the function of TLR4 in hepatoma cells. The ligand (LPS) of TLR4 activates the STAT3 pathway by activating the COX-2/PGE2 signal axis, resulting in proliferation of liver cancer cells [135]. Liver cancer patients have characteristics of intestinal flora imbalance and intestinal wall permeability increase. Once the intestinal flora is translocated to the liver, its metabolites (LPS) activate TLR4 in orthotopic liver cells to promote inflammation. This suggests that in the treatment of clinical liver cancer, improving intestinal flora imbalance may be used as an adjuvant treatment for HCC treatment and prognosis.

Numerous evidences suggest that intestinal flora is associated with oncogene signaling, and the clarification of causality is increasingly contributing to the development of HCC. The regulation of intestinal flora by antibiotics and probiotics may be a new strategy to prevent the development of chronic hepatitis, cirrhosis and HCC. However, the role of intestinal flora in the process of HCC is still unclear. Further research into the composition of gut flora is needed for long-term stability in human health and disease.

## 9. Major Therapeutic Strategies for Hepatocarcinogenesis

In recent years, molecular targeted therapy for liver cancer has received extensive attention. The clarification of the above molecular mechanisms has allowed the integration of research and clinical treatment to provide effective treatment to patients. At present, over 100 kinds of small molecule target drugs are in phase I–IV of clinical research. However, small molecule drugs against tumors are not yet effective for all patients with liver cancer; moreover, the extreme complication of the regulatory mechanisms further result in the limited effect of single drugs against cancer. Therefore, the search for specific tumor markers will likely lead to markedly improved clinical treatment efficacy.

### 9.1. Anti-Angiogenesis 

The VEGF pathway is transduced by tyrosine kinases, such as VEGFR1, VEGFR2, and VEGFR3 and is essential for tumorigenesis and development. The VEGF pathway enhances mitosis of vascular endothelial cells, lymphatic endothelial cells, monocytes, and macrophages, stimulates cell proliferation and capillary angiogenesis, enhances microvascular permeability, and promotes the deposition of extravascular matrix, providing nutrition for tumor vessels. VEGFR2 has been shown to be of therapeutic value for most solid tumors. Although studies have reported that some clinical pathological features of HCC are related to the expression and expression regions of VEGFR2, the results are still unsatisfactory. Drugs that target angiogenesis include VEGFRs that can be divided into three classes: VEGF monoclonal antibodies, VEGFRs, and small molecule kinase inhibitors. The targeted drugs associated with this pathway are as follows:

#### 9.1.1. Bevacizumab

Bevacizumab is a monoclonal antibody that binds to circulating VEGF as measured through in vitro and in vivo detection systems, and prevents its binding to endothelial cell surface receptors, thereby inhibiting microangiogenesis. Consistent with this, bevacizumab also regulates tumor microvessel density and is normally distributed in surrounding tissue vessels. Previous studies have shown that bevacizumab is effective against tumors and can enhance the delivery of chemotherapy drugs. Bevacizumab can affect tumor growth by inhibiting angiogenesis, in addition to affecting regulation by inhibiting autocrine VEGFR to further promote the apoptosis and inhibition of proliferation of tumor cells, thereby improving radiotherapy and chemotherapy sensitivity [136]. Recently, the Food and Drug Administration (FDA) approved the use of bevacizumab in the clinical treatment of various malignant tumors such as ovarian cancer, colon cancer, kidney cancer, and lung cancer. Notably, clinical studies have shown that the expression level of VEGFR2 can affect its therapeutic effect on patients [137], which is of great significance for future studies. However, at present, the FDA has not yet approved bevacizumab for the treatment of HCC. A number of phase II clinical studies have explored the efficacy of bevacizumab alone and in combination with chemotherapy on HCC. The preliminary results show that the efficacy is not obvious, with treatment being prone to causing bleeding and other adverse reactions. The exact role of this HCC therapy thus remains to be further validated in a phase III clinical study of big data [138].

#### 9.1.2. Sunitinib

Sunitinib constitutes an inhibitor of tyrosine kinase dual receptors that targets PDFGR, VEGFR, and FLT3. The experimental results show that its inhibitory effect on angiogenesis is stronger than that of sorafenib, a tyrosine kinase inhibitor utilized against Ras/Raf/Mek/Erk signaling (described below), and its target of action is closely related to angiogenesis. Sunitinib inhibits the activity of these targets and influences signal transduction. It can further inhibit tumor angiogenesis and achieve anti-tumor effects. Although sunitinib exhibits certain anti-HCC activity, most patients with HCC have a medical history of liver disease and have worse liver function, poor tolerance, and high incidence of adverse reactions with this treatment [139,140].

#### 9.1.3. Apatinib

Apatinib is a derivative of the small molecule VEGFR tyrosine kinase inhibitor PTK787 and constitutes the first multi-target, safe, and effective therapy for advanced refractory gastric cancer. Apatinib targets include VEGFR1, VEGFR2, c-Kit, c-Src, and PDGFR, which can specifically inhibit the activity of VEGFR2, block the binding of VEGF to the receptor as well as downstream signal transduction; inhibit neovascularization; reduce tumor vascular density; and inhibit tumor growth [141,142,143].

#### 9.1.4. Linifanib

Linifanib constitutes a novel ATP competitive inhibitor that is highly selective for VEGF and PDGF receptors, but has no significant effect on cytoplasmic tyrosine kinase and serine/threonine kinase. Its anti-vascular activity has been explored in numerous clinical trials. Preclinical and early-stage trials have exhibited positive results, and antitumor activity has been demonstrated in many studies through its effect on signaling pathway targets. The problems faced by the development of linifanib in later stages comprise of limited efficacy and drug toxicity. Thus, strategies to reduce drug toxicity and identify biomarkers represent goals for the further development of linifanib [144,145].

#### 9.1.5. Thalidomide

Thalidomide is used in multiple myeloma, in which it may exert immunoregulatory, immunosuppressive, anti-inflammatory, and neutrophil chemotaxis inhibitory functions. The drug exhibits a close relationship with TNF-α through which it exerts an anti-angiogenic effect by modulating the secretion of other cytokines such as fibroblast growth factor and VEGF [146]. Studies have found that thalidomide can inhibit angiogenesis by this mechanism, as well as by inhibiting the synthesis of integrin on the surface of leukocytes [147]. Previous studies have indicated that thalidomide combined with transarterial chemoembolization (TACE) in the treatment of HCC, using clinical efficacy and clinical adverse reactions as indicators, demonstrated increased productivity in the treatment group at six months and one year, although the difference was not statistically significant. Moreover, although the AFP in the treatment group decreased, the difference was not statistically significant, suggesting that thalidomide combination therapy did not achieve satisfactory results, and there was no evidence that it was advantageous [148].

### 9.2. Ras/Raf/Mek/Erk Signaling

The Ras/Raf/Mek/Erk signaling pathway comprises of a chain of protein signal pathways that are transported layer-by-layer through the cell receptor to the nuclear DNA. The pathway begins with signal molecule binding to receptors on the cell surface, continuing through signaling in the nucleus to initiate gene transcription and protein expression, eventually causing a series of changes, such as cell division. Sorafenib reduces the activity of Raf-1 and B-Raf serine/threonine kinases, inhibits the activity of tyrosine kinase receptors in VEGFRs, and regulates the intervention of PDGFR-b, Ret, and c-Kit, which inhibit tumor growth and angiogenesis [149,150,151]. A multi-center international comparison of sorafenib in the United States and Europe in Phase III clinical studies of HCC has confirmed that sorafenib significantly prolongs the survival of patients and delays the progression of tumors [152].

### 9.3. PI3K/Akt/mTOR Signaling

The PI3K/Akt/mTOR signaling pathway plays an important role in the occurrence and development of liver cancer. Among its components, mTOR downstream of PI3K/Akt acts as a key kinase in this pathway, which mainly regulates the biological effects of tumor cell proliferation, growth, survival, and angiogenesis [153]. The main mechanism of this pathway is to inhibit apoptosis-related factors and activate anti-apoptotic factors by activating phosphorylated PI3K/Akt/mTOR signaling pathways. The phosphorylated tumor suppressor gene PTEN negatively regulates PI3K, whereas low expression of PTEN activates the PI3K/Akt/mTOR pathway leading to tumor formation. Recent studies have shown that this pathway plays an important role in tumor cell self-renewal and constitutes one of the major causes of tumor recurrence and metastasis [154].

#### 9.3.1. Sirolimus

Sirolimus (rapamycin) is a potent immunosuppressive agent that inhibits graft-versus-host rejection after liver transplantation, and has recently been shown to down-regulate the expression of hypoxia-inducible factor-1α (HIF-1α) by inhibiting mTOR. Reducing the synthesis and secretion of VEGF by sirolimus has been shown to effectively inhibit the growth of liver cancer [155]. Moreover, a Phase III clinical trial has demonstrated that rapamycin is effective as a basic immunotherapy after hepatectomy [156].

#### 9.3.2. Everolimus

Everolimus constitutes a therapeutic drug that has been successively approved by the United States FDA for advanced renal cell carcinoma, breast cancer, and pancreatic neuroendocrine tumors. A phase II study of HCC demonstrated the efficacy of everolimus. However, the results of a phase III clinical trial of sorafenib (EVOLVE-1) showed that everolimus yields no significant improvement in the prognosis of patients with HCC who are refractory or intolerable to sorafenib treatment. The combination with sorafenib also failed to achieve the desired effect [157].

### 9.4. Wnt/β-Catenin Signaling

In the Wnt/β-catenin signaling pathway, Wnt proteins bind to transmembrane protein receptors upon stimulation. Subsequently, glycogen synthase kinase 3β (GSK-3β) is inhibited, which in turn leads to stable nuclear translocation of β-catenin. Beta-catenin, in combination with transcription factors of the T-cell family, activates numerous oncogenes including Cyclin D1 and proto-oncogenes in addition to stimulating the transcription of proto-oncogene mRNA, promoting cancer cell survival and proliferation. Studies have shown that there is accumulation of β-catenin in 50% to 70% of liver cancer specimens [158]. Accordingly, previous clinical studies targeted interference with β-catenin, such as via PKF115-845 and CGP049090, which can block the binding of β-catenin and TCF and inhibit the subsequent transcriptional co-activation [159].

### 9.5. NF-κB Signaling

NF-κB constitutes an important nucleoprotein factor that exists in the cytoplasm and plays an important role in the development and progression of immunity, inflammation, and tumors by regulating cell proliferation, differentiation, apoptosis, and metastasis. Studies have found that NF-κB is activated in tumors and can promote the growth and differentiation of tumor cells. Its degree of activity is related to the ability of tumors to metastasize. In addition, some cytokines involved in the process of tumor cell growth are mediated by NF-κB [160]. The mechanism of action of bortezomib is to inhibit the decomposition of NF-κB-activated proteins in cells, inhibit downstream information transmission, trigger apoptosis, and increase the sensitivity of liver cancer cells to chemotherapeutic drugs. Consistent with this, an in vitro study has shown that bortezomib exhibits a cytotoxic effect on hepatocellular carcinoma cells [161]. Phase II clinical trials have shown that bortezomib has a certain effect on advanced liver cancer, pending further data from clinical trials that are necessary to complete the follow-up study [162].

### 9.6. Immunotargeting Drugs

Tremelimumab is a fully humanized monoclonal antibody against cytotoxic T lymphocyte-associated antigen 4 (CTLA-4) that blocks the signaling pathways used to assist tumors in escaping immune surveillance. Tremelimumab stimulates the immune system to attack tumor cells by binding CTLA-4 protein expressed on the surface of activated T lymphocytes [163]. In a Phase II study, 20 patients with advanced HCC with HCV were treated with tremelimumab after systemic failure by agents such as sorafenib, and observed for anti-tumor and anti-HCV activity. The results showed that the partial remission rate was 17.6%, the disease control rate was 76.4%, the median time to progression was 6.48 months, and safety was good. Only transient transaminase elevation occurred in the first cycle, which warrants further study [164].

### 9.7. Inflammasome-Targeting Therapy

Inhibitors for targeting inflammasome activation, especially in NLRP3 inflammasomes, are currently attracting considerable attention. In the pathogenesis of HCC, several pharmacological inhibitors of the inflammasome signaling cascade have been investigated, such as Parthenolide Bay 11­7082, isoliquiritigenin (ILG), sulforaphane (SFN), two liver X receptors T0901317 and GW3965, and Spirodalesol. The parthenolide and Bay 11-7082, an herbal NF-κB inhibitor and synthetic IκB kinase-β inhibitor, directly inhibited the expression of the NLRP3 inflammasome by inhibiting ATPase activity, leading to significantly decreased activity of caspase-1 [165]. ILG-mediated suppression of NLRP3 and down-regulation of caspase-1 and IL-1β activation has also been reported [166]. Similar results were reported for SFN, two liver X receptors T0901317 and GW3965, and Spirodalesol [167,168,169]. Recently, the ketone body β-hydroxybutyrate (BHB) and MCC950 were revealed to specifically inhibit inflammasome activation. BHB serves as an alternative source of ATP when cells are under an energy deficit status. The inhibitory activity of BHB was noted in NLRP3-mediated diseases including Muckle-Wells syndrome and familial cold autoinflammatory syndrome, by reducing caspase-1 activation and IL-1β secretion through the reduction of potassium efflux and ASC oligomerization [170]. MCC950 represents a new inhibitor of the NLRP3 inflammasome, which acts by inhibiting both canonical and noncanonical NLRP3-dependent inflammasomes [171,172,173,174]. In multiple sclerosis, MCC950 significantly reduces the release of IL-1β by blocking NLRP3 inflammasomes [171]. However, the detailed mechanism of NLRP3 inhibition by MCC950 is not clear. Further data are necessary to confirm these findings and provide a strong foundation toward therapeutic application of this drug.

### 9.8. Immunotherapy for HCC

In recent years, tumor immunotherapy is being seen as a promising method for inhibiting tumor progression, a novel treatment strategy that may lead to improvements in outcome. Here, we briefly list the major immunotherapeutic approaches with their current advances in HCC (Table 1). The five major immunotherapeutic approaches for HCC have their own characteristics and functions: (1) Cytokine therapy plays an important role in the treatment of liver cancer and prevention of recurrence. It not only has an antiviral effect, but also enhances immunity, exerts anti-tumor angiogenesis, induces cell differentiation and apoptosis, and inhibits tumor cell proliferation; (2) The cell transfer immunotherapy works by expanding the patient’s own lymphocytes ex vivo and then reinfusing into the patient; (3) Immune checkpoint inhibitors can reactivate tumor-specific T cells and develop an antitumor effect by suppressing checkpoint-mediated signaling. Immune checkpoint receptors are often upregulated in tumor tissue and promote tumor evasion from host immunosurveillance; (4) Cancer vaccination is performed using effector T cells to activate tumor-specific immune responses that can specifically decrease tumor load and control tumor relapse; (5) Indoleamine 2,3-dioxygenase (IDO), which degrades L-tryptophan along the kynurenine pathway, suppresses CD4+ T-cell function. 

## 10. Conclusions and Future Directions

In summary, the focus of future studies in HCC is the research and development of combined therapies involving multi-molecule, multi-channel targeted drug combination therapy strategies. Antibody-, receptor- and vector-mediated liver-targeted drug delivery systems optimize drug delivery and efficacy and reduce drug dose and adverse drug reactions, and may be actively applied to specific individuals targeted through individual differences and genetic polymorphisms. Molecularly targeted drugs have been used to establish an effective classification system for liver cancer molecular abnormalities, with specific populations being targeted for treatment through the use of biomolecules, enzymes, and gene expression data. Through standard clinical trials, patients with specific tumors can thus be provided with the best individualized treatment plan. It is expected that in the near future, the treatment of liver cancer can achieve precise targeted therapy.

## Figures and Tables

**Table 1:** Potentiality of immunotherapy against HCC.

Strategies	Subsets	Targets and Applications	Refs.
Cytokine therapy	IFN IL-2	INF-α2b, INF-β, INF-α, INF-α + 5-FU, IFN-γ + GM-CSF	[175,176,177,178,179,180]
Cell transfer immunotherapy	CIK cells NK cells CAR T cells	CIK with RFA and/or TACE, TAA-pulsed DC and CIK RFA + NK, sorafenib, NKG2D GPC3, EpCAM, VEGF-A + Osteopontin	[181,182,183,184,185,186]
Immune checkpoint inhibitors	PD-1 inhibitors PDL-1 inhibitors CTL-A4 inhibitors	Nivolumab, Decitabine, c-Met inhibitor, Pidilizumab Durvalumab, Tremelimumab, Ipilimumab, DNMT1 inhibitor Anti-CTL-A4 antibody, Tremelimumab with RFA or TACE	[164,187,188,189,190,191,192,193,194,195,196]
Vaccine strategy	Antigen peptide vaccines DC vaccines	AFP, GPC3, NY-ESO-1, SSX-2, HCA587, MAGE-A3, TERT DC + CIK, DC + radiation, DC + AFP peptide	[197,198,199,200,201]
IDO inhibitor	Indoximod	L-tryptophan (Trp) into L-kynurenine (Kyn)	[202]

CTL-A4: Cytotoxic T-Lymphocyte Associated Protein 4; PD-1: Programmed cell death-1; PDL-1: Programmed death-ligand 1; RFA: radiofrequency ablation; TACE: transarterial chemoembolization; GPC3: αFP and glypican-3; CIK: cytokine induced killer; TILs: tumor infiltrating lymphocytes; NK: Natural killer; CAR T: Chimeric antigen receptor T cell; IDO: indoleamine 2,3-dioxygenase; TAA: Targeting of tumor-associated antigens, INF: interferon.
